# 10-Formyl-2,4,6,8,12-penta­nitro-2,4,6,8,10,12-hexa­azatetra­cyclo­[5.5.0.0^3,11^.0^5,9^]dodeca­ne

**DOI:** 10.1107/S1600536809047138

**Published:** 2009-11-18

**Authors:** Shaohua Jin, Shusen Chen, Huaxiong Chen, Lijie Li, Yanshan Shi

**Affiliations:** a School of Materials Science and Engineering, Beijing Institute of Technology, Beijing 100081, People’s Republic of China

## Abstract

The title compound, C_7_H_7_N_11_O_11_ (PNMFIW), is a caged heterocycle substituted with five nitro and one formyl groups. It is related to the hexa­azaisowurtzitane family of high-density high-energy polycyclic cage compounds. Four nitro groups are appended to the four N atoms of the two five-membered rings, while a nitro group and a formyl are attached to the two N atoms of the six-membered ring.

## Related literature

The title compound (PNMFIW) was reported as a by-product in the synthesis of hexa­nitro­hexa­azawurtzitane (HNIW), see: Golfier *et al.* (1998 ▶). Liu *et al.* (2006 ▶). For quantum calculations on HNIW and PNMFIW, see: Wu *et al.* (2003 ▶). For factors affecting the detonation performance of energetic compounds, see: Singh & Felix (2003 ▶); Zeman & Krupka (2003 ▶).
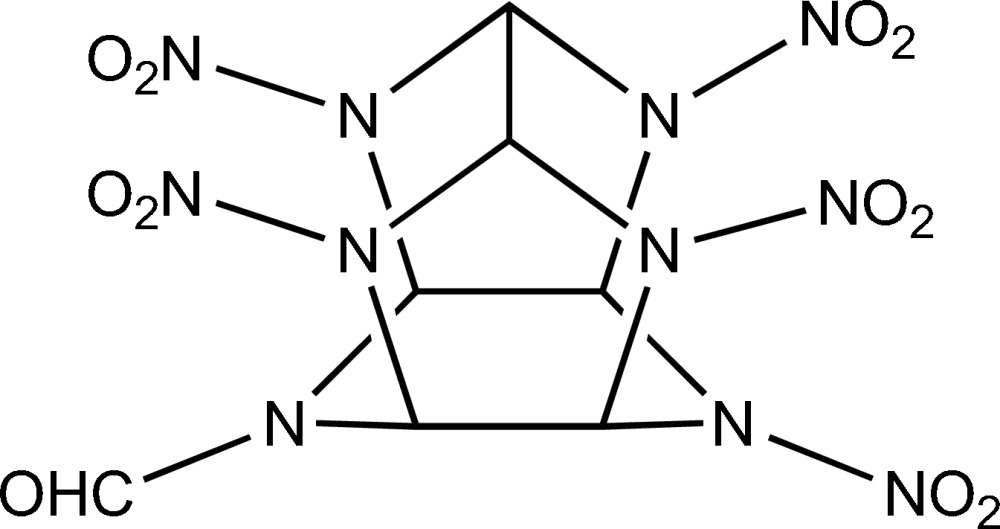


## Experimental

### 

#### Crystal data

C_7_H_7_N_11_O_11_
*M* *_r_* = 421.24
Orthorhombic, 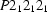

*a* = 8.8000 (18) Å
*b* = 12.534 (2) Å
*c* = 12.829 (3) Å
*V* = 1415.1 (5) Å^3^
*Z* = 4
Mo *K*α radiation
μ = 0.19 mm^−1^
*T* = 93 K
0.33 × 0.30 × 0.20 mm

#### Data collection

Rigaku Saturn724+ diffractometer
Absorption correction: none
11499 measured reflections
1865 independent reflections
1773 reflections with *I* > 2σ(*I*)
*R*_int_ = 0.036

#### Refinement

*R*[*F* ^2^ > 2σ(*F* ^2^)] = 0.034
*wR*(*F* ^2^) = 0.084
*S* = 1.00
1865 reflections
263 parameters
H-atom parameters constrained
Δρ_max_ = 0.36 e Å^−3^
Δρ_min_ = −0.27 e Å^−3^

### 

Data collection: *CrystalClear* (Rigaku, 2002 ▶); cell refinement: *CrystalClear*; data reduction: *CrystalClear*; program(s) used to solve structure: *SHELXS97* (Sheldrick, 2008 ▶); program(s) used to refine structure: *SHELXL97* (Sheldrick, 2008 ▶); molecular graphics: *SHELXTL* (Sheldrick, 2008 ▶); software used to prepare material for publication: *SHELXL97*.

## Supplementary Material

Crystal structure: contains datablocks I, global. DOI: 10.1107/S1600536809047138/nk2008sup1.cif

Structure factors: contains datablocks I. DOI: 10.1107/S1600536809047138/nk2008Isup2.hkl

Additional supplementary materials:  crystallographic information; 3D view; checkCIF report

## Figures and Tables

**Fig. 1.**
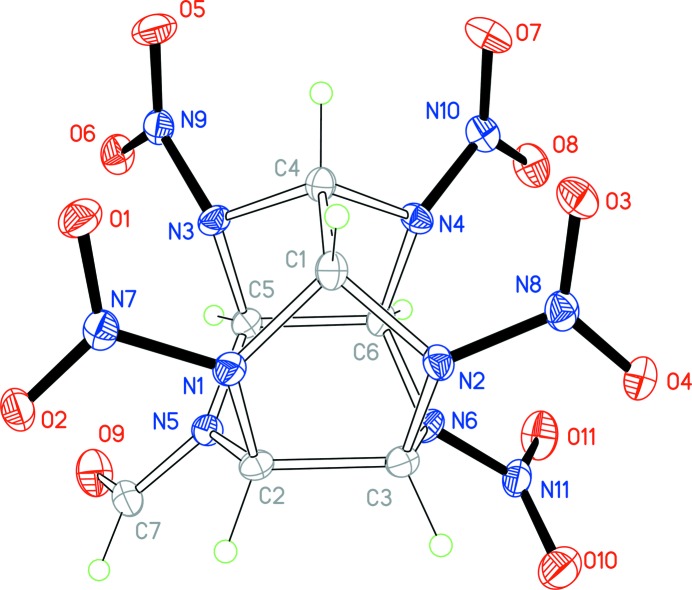
The structure of PNMFIW with displacement ellipsoids drawn at the 50% probability level.

## supplementary crystallographic information

### Comment

The title compound, pentanitromonoformylhexaazaisowurtzitane (PNMFIW), was reported as a by-product in the synthesis of hexanitrohexaazawurtzitane (HNIW) (Golfier *et al.*, 1998; Liu *et al.*,2006). We theoretically studied PNMFIW with quantum calculation and found that PNMFIW has a similar crystal structure and density but lower sensitivity compared with HNIW (Wu *et al.*, 2003). The crystal structure of an energetic compound is very important, because detonation performance such as detonation velocity and pressure, depends largely on density which is identified by its crystal structure, while the sensitivity closely correlates with the crystal structure (Singh *et al.*, 2003; Zeman *et al.*, 2003). To now, the crystal structure and properties of PNMFIW have not been reported. We synthesized PNMFIW through the nitrolysis of tetraacetyldiformylhexaazaisowurtzitane (TADFIW) in mixed nitric and sulfuric acids and obtained single crystals of PNMFIW by controlled evaporation.

The main geometric parameters of PNMFIW are listed in Tables 1 and 2, and the molecular structure is illustrated in Fig.1. The cage structure of PNMFIW is constructed from one six-membered and two five-membered rings which are closed by the C1—C4 bond, thus creating two seven-membered rings. The six-membered pyrazine ring in PNMFIW boat-shaped , while more stable conformation of six-membered ring is in the chair form. The two five-membered rings are also non-planar, being characterized by the torsion angles of two five-membered rings. Four nitro groups are appended to the four nitrogen atoms of the two five-membered rings, while a nitro group and a formyl are attached to the two nitrogen atoms of the six-membered ring respectively. Due to cage structure of PNMFIW the bond length of N—N (1.369–1.436 Å) is much longer than common nitramine (1.360 Å). The bond length of C—C (1.561–1.587 Å) of PNMFIW is also much longer than common C—C bond (1.54 Å). Bond angles of N(4)—C(5)—C(6) (112.7°), C(7)—N(1)—C(1)(122.2°) and C(2)—N(2)—C(3) (117.5°) on caged structure are much bigger than normal angle of *sp*^3^ hybrid bond. From the molecular structure analysis above, we know that PNMFIW molecule has high tensile force and energy.

### Experimental

Fuming sulfuric acid was slowly added into fuming nitric acid in a three-neck flask with stirring. After the solution of mixed acids was heated to 60 °C, tetraacetyldiformylhexaazaisowurtzitane (10 g) was added, and then the temperature was elevated to 65 °C. The solution was maintained at 65 °C for 12 h; thereafter the solution was poured into ice-water. The precipitated solid was filtered off, washed with water and then dried. The obtained solid was a mixture of polynitrohexaazaisowurtzitane derivatives with different number of nitro substitutes. Pure PNMFIW was obtained through a silica column chromatography with hexane/acetyl acetate (6/4 by volume) as mobile phase at room temperature (25 °C).

Pure PNMFIW was dissolved in mixed solvents of acetone and n-hexane, and then the resulted solution was placed in ambient condition (288–293 K). A week later, single crystals was obtained by controlling the evaporation of solvent. Elemental analysis, FT—IR, MS and ^1^H NMR are in agreement with the structure of PNMFIW.

### Refinement

All non-hydrogen atoms were obtained from the difference Fourier map and refined with atomic anisotropic thermal parameters. The hydrogen atoms were placed geometrically and treated a constrained refinement. All C–H distances are constrained to 1.00 Å, except C7—H7 which is 0.95 Å. In all cases *U*_eq_(H) = 1.2*U*_eq_(C). Friedel pairs were merged during final refinement owing to the lack of anomalous dispersion data.

### Crystal data

**Table:**

C_7_H_7_N_11_O_11_	*D*_x_ = 1.977 Mg m^−^^3^
*M**_r_* = 421.24	Melting point: 247 K
Orthorhombic, *P*2_1_2_1_2_1_	Mo *K*α radiation, λ = 0.71073 Å
Hall symbol: P 2ac 2ab	Cell parameters from 5010 reflections
*a* = 8.8000 (18) Å	θ = 3.2–27.5°
*b* = 12.534 (2) Å	µ = 0.19 mm^−^^1^
*c* = 12.829 (3) Å	*T* = 93 K
*V* = 1415.1 (5) Å^3^	Prism, colourless
*Z* = 4	0.33 × 0.30 × 0.20 mm
*F*(000) = 856	

### Data collection

**Table:**

Rigaku Saturn724+ diffractometer	1773 reflections with *I* > 2σ(*I*)
Radiation source: Rotating Anode	*R*_int_ = 0.036
graphite	θ_max_ = 27.5°, θ_min_ = 3.2°
Detector resolution: 28.5714 pixels mm^-1^	*h* = −11→10
Multi–scan	*k* = −16→14
11499 measured reflections	*l* = −16→16
1865 independent reflections	

### Refinement

**Table:**

Refinement on *F*^2^	Secondary atom site location: difference Fourier map
Least-squares matrix: full	Hydrogen site location: inferred from neighbouring sites
*R*[*F*^2^ > 2σ(*F*^2^)] = 0.034	H-atom parameters constrained
*wR*(*F*^2^) = 0.084	*w* = 1/[σ^2^(*F*_o_^2^) + (0.0486*P*)^2^ + 0.596*P*] where *P* = (*F*_o_^2^ + 2*F*_c_^2^)/3
*S* = 1.00	(Δ/σ)_max_ = 0.001
1865 reflections	Δρ_max_ = 0.36 e Å^−^^3^
263 parameters	Δρ_min_ = −0.26 e Å^−^^3^
0 restraints	Extinction correction: *SHELXL97* (Sheldrick, 2008), Fc^*^=kFc[1+0.001xFc^2^λ^3^/sin(2θ)]^-1/4^
10 constraints	Extinction coefficient: 0.0821 (10)
Primary atom site location: structure-invariant direct methods	

### Special details

**Table:**

Geometry. All e.s.d.'s (except the e.s.d. in the dihedral angle between two l.s. planes) are estimated using the full covariance matrix. The cell e.s.d.'s are taken into account individually in the estimation of e.s.d.'s in distances, angles and torsion angles; correlations between e.s.d.'s in cell parameters are only used when they are defined by crystal symmetry. An approximate (isotropic) treatment of cell e.s.d.'s is used for estimating e.s.d.'s involving l.s. planes.
Refinement. Refinement of *F*^2^ against ALL reflections. The weighted *R*-factor *wR* and goodness of fit *S* are based on *F*^2^, conventional *R*-factors *R* are based on *F*, with *F* set to zero for negative *F*^2^. The threshold expression of *F*^2^ > σ(*F*^2^) is used only for calculating *R*-factors(gt) *etc*. and is not relevant to the choice of reflections for refinement. *R*-factors based on *F*^2^ are statistically about twice as large as those based on *F*, and *R*- factors based on ALL data will be even larger.

### Fractional atomic coordinates and isotropic or equivalent isotropic displacement parameters (Å^2^)

**Table:**

	*x*	*y*	*z*	*U*_iso_*/*U*_eq_	
O1	0.4007 (2)	0.13038 (16)	0.12911 (15)	0.0191 (4)	
O2	0.3409 (2)	0.08809 (15)	0.28944 (15)	0.0179 (4)	
O3	0.1006 (2)	0.45094 (16)	0.01761 (14)	0.0191 (4)	
O4	−0.0499 (2)	0.50366 (16)	0.14359 (15)	0.0199 (4)	
O5	0.6743 (2)	0.34574 (17)	0.13169 (16)	0.0222 (5)	
O6	0.6994 (2)	0.37181 (16)	0.29844 (16)	0.0202 (4)	
O7	0.4174 (2)	0.56954 (17)	0.04576 (14)	0.0223 (5)	
O8	0.3477 (2)	0.66003 (15)	0.18230 (16)	0.0216 (5)	
O9	0.4369 (3)	0.28833 (17)	0.52530 (16)	0.0239 (5)	
O10	−0.0814 (2)	0.51961 (18)	0.37613 (17)	0.0265 (5)	
O11	0.1111 (2)	0.62630 (16)	0.38019 (17)	0.0248 (5)	
N1	0.2231 (3)	0.22215 (17)	0.21233 (17)	0.0130 (4)	
N2	0.0880 (2)	0.35693 (18)	0.16357 (16)	0.0126 (5)	
N3	0.4655 (2)	0.34315 (18)	0.23254 (17)	0.0132 (5)	
N4	0.3187 (3)	0.48471 (17)	0.18203 (17)	0.0128 (4)	
N5	0.3024 (2)	0.30228 (17)	0.37498 (17)	0.0126 (4)	
N6	0.1429 (3)	0.45977 (17)	0.32082 (17)	0.0135 (5)	
N7	0.3299 (3)	0.14247 (17)	0.21021 (18)	0.0148 (5)	
N8	0.0428 (3)	0.44415 (18)	0.10471 (17)	0.0155 (5)	
N9	0.6262 (3)	0.35696 (18)	0.21936 (19)	0.0162 (5)	
N10	0.3673 (3)	0.57783 (18)	0.13405 (18)	0.0158 (5)	
N11	0.0508 (3)	0.54120 (18)	0.36035 (18)	0.0173 (5)	
C1	0.2300 (3)	0.3052 (2)	0.1333 (2)	0.0128 (5)	
H1	0.2235	0.2746	0.0614	0.015*	
C2	0.1756 (3)	0.2665 (2)	0.31371 (19)	0.0124 (5)	
H2	0.1133	0.2136	0.3534	0.015*	
C3	0.0756 (3)	0.3640 (2)	0.27868 (19)	0.0132 (5)	
H3	−0.0321	0.3556	0.3021	0.016*	
C4	0.3729 (3)	0.3812 (2)	0.1456 (2)	0.0130 (5)	
H4	0.4327	0.3871	0.0795	0.016*	
C5	0.3971 (3)	0.3834 (2)	0.33015 (19)	0.0122 (5)	
H5	0.4779	0.4058	0.3804	0.015*	
C6	0.2991 (3)	0.4819 (2)	0.29537 (19)	0.0126 (5)	
H6	0.3352	0.5493	0.3288	0.015*	
C7	0.3332 (3)	0.2587 (2)	0.4713 (2)	0.0160 (6)	
H7	0.2699	0.2028	0.4962	0.019*	

### Atomic displacement parameters (Å^2^)

**Table:**

	*U*^11^	*U*^22^	*U*^33^	*U*^12^	*U*^13^	*U*^23^
O1	0.0186 (10)	0.0190 (10)	0.0197 (9)	0.0025 (8)	0.0057 (8)	−0.0027 (8)
O2	0.0180 (10)	0.0136 (9)	0.0221 (9)	0.0009 (8)	−0.0018 (8)	0.0041 (8)
O3	0.0227 (11)	0.0224 (10)	0.0121 (8)	−0.0001 (9)	−0.0025 (8)	0.0020 (7)
O4	0.0164 (9)	0.0197 (10)	0.0237 (10)	0.0058 (8)	−0.0005 (9)	0.0022 (8)
O5	0.0185 (10)	0.0264 (11)	0.0217 (10)	−0.0001 (9)	0.0084 (9)	−0.0004 (9)
O6	0.0128 (9)	0.0199 (10)	0.0278 (11)	−0.0016 (8)	−0.0045 (8)	0.0039 (9)
O7	0.0298 (12)	0.0221 (10)	0.0150 (9)	−0.0062 (10)	0.0022 (8)	0.0041 (8)
O8	0.0288 (11)	0.0113 (9)	0.0247 (10)	−0.0010 (8)	−0.0034 (9)	−0.0003 (8)
O9	0.0279 (11)	0.0241 (11)	0.0196 (10)	0.0030 (10)	−0.0095 (9)	−0.0009 (8)
O10	0.0213 (11)	0.0316 (12)	0.0267 (10)	0.0032 (9)	0.0019 (9)	−0.0012 (10)
O11	0.0291 (12)	0.0140 (10)	0.0312 (11)	0.0037 (9)	−0.0051 (10)	−0.0073 (9)
N1	0.0127 (10)	0.0120 (10)	0.0142 (10)	0.0011 (9)	0.0016 (9)	−0.0004 (9)
N2	0.0143 (11)	0.0129 (11)	0.0105 (10)	0.0017 (9)	−0.0015 (8)	0.0006 (8)
N3	0.0081 (10)	0.0152 (11)	0.0161 (11)	−0.0009 (9)	0.0007 (8)	0.0000 (9)
N4	0.0164 (11)	0.0089 (10)	0.0132 (10)	−0.0019 (9)	0.0011 (9)	0.0007 (8)
N5	0.0113 (10)	0.0144 (11)	0.0123 (10)	−0.0008 (9)	−0.0014 (9)	0.0006 (8)
N6	0.0122 (11)	0.0132 (11)	0.0152 (10)	0.0015 (9)	0.0007 (9)	−0.0045 (9)
N7	0.0134 (11)	0.0111 (10)	0.0198 (11)	−0.0014 (9)	−0.0012 (9)	−0.0012 (9)
N8	0.0137 (11)	0.0167 (11)	0.0160 (11)	−0.0002 (9)	−0.0039 (9)	0.0012 (9)
N9	0.0117 (10)	0.0126 (11)	0.0242 (11)	−0.0011 (9)	0.0003 (10)	0.0038 (10)
N10	0.0174 (11)	0.0141 (11)	0.0159 (10)	−0.0025 (9)	−0.0029 (10)	0.0006 (9)
N11	0.0162 (11)	0.0187 (12)	0.0171 (11)	0.0058 (10)	−0.0012 (10)	0.0003 (9)
C1	0.0126 (12)	0.0125 (12)	0.0131 (11)	0.0003 (10)	−0.0036 (10)	−0.0011 (10)
C2	0.0121 (12)	0.0145 (12)	0.0107 (11)	0.0001 (10)	0.0015 (9)	−0.0018 (10)
C3	0.0121 (12)	0.0146 (12)	0.0129 (11)	0.0000 (10)	0.0011 (10)	−0.0003 (10)
C4	0.0117 (12)	0.0133 (12)	0.0139 (11)	0.0007 (10)	−0.0015 (10)	0.0001 (10)
C5	0.0108 (12)	0.0132 (12)	0.0126 (11)	−0.0007 (10)	−0.0008 (10)	−0.0011 (9)
C6	0.0119 (12)	0.0132 (12)	0.0127 (11)	0.0014 (10)	−0.0021 (10)	−0.0025 (10)
C7	0.0194 (14)	0.0139 (13)	0.0146 (12)	0.0015 (11)	−0.0012 (11)	0.0011 (11)

### Geometric parameters (Å, °)

**Table:**

O1—N7	1.222 (3)	N4—N10	1.387 (3)
O2—N7	1.228 (3)	N4—C4	1.460 (3)
O3—N8	1.231 (3)	N4—C6	1.465 (3)
O4—N8	1.212 (3)	N5—C7	1.378 (3)
O5—N9	1.210 (3)	N5—C5	1.435 (3)
O6—N9	1.216 (3)	N5—C2	1.436 (3)
O7—N10	1.220 (3)	N6—N11	1.398 (3)
O8—N10	1.214 (3)	N6—C6	1.440 (3)
O9—C7	1.205 (3)	N6—C3	1.444 (3)
O10—N11	1.211 (3)	C1—C4	1.586 (3)
O11—N11	1.218 (3)	C1—H1	1.0000
N1—N7	1.372 (3)	C2—C3	1.571 (4)
N1—C1	1.454 (3)	C2—H2	1.0000
N1—C2	1.475 (3)	C3—H3	1.0000
N2—N8	1.387 (3)	C4—H4	1.0000
N2—C1	1.460 (3)	C5—C6	1.570 (4)
N2—C3	1.483 (3)	C5—H5	1.0000
N3—N9	1.435 (3)	C6—H6	1.0000
N3—C4	1.461 (3)	C7—H7	0.9500
N3—C5	1.478 (3)		
			
N7—N1—C1	118.6 (2)	N1—C1—H1	111.5
N7—N1—C2	119.1 (2)	N2—C1—H1	111.5
C1—N1—C2	110.9 (2)	C4—C1—H1	111.5
N8—N2—C1	116.8 (2)	N5—C2—N1	112.4 (2)
N8—N2—C3	118.3 (2)	N5—C2—C3	110.4 (2)
C1—N2—C3	110.7 (2)	N1—C2—C3	101.50 (19)
N9—N3—C4	114.8 (2)	N5—C2—H2	110.7
N9—N3—C5	117.4 (2)	N1—C2—H2	110.7
C4—N3—C5	108.0 (2)	C3—C2—H2	110.7
N10—N4—C4	120.3 (2)	N6—C3—N2	113.1 (2)
N10—N4—C6	119.8 (2)	N6—C3—C2	108.1 (2)
C4—N4—C6	109.6 (2)	N2—C3—C2	101.38 (19)
C7—N5—C5	121.7 (2)	N6—C3—H3	111.3
C7—N5—C2	121.3 (2)	N2—C3—H3	111.3
C5—N5—C2	116.9 (2)	C2—C3—H3	111.3
N11—N6—C6	119.7 (2)	N4—C4—N3	103.1 (2)
N11—N6—C3	120.4 (2)	N4—C4—C1	107.9 (2)
C6—N6—C3	117.8 (2)	N3—C4—C1	108.8 (2)
O1—N7—O2	126.6 (2)	N4—C4—H4	112.2
O1—N7—N1	117.2 (2)	N3—C4—H4	112.2
O2—N7—N1	116.2 (2)	C1—C4—H4	112.2
O4—N8—O3	127.5 (2)	N5—C5—N3	109.5 (2)
O4—N8—N2	117.0 (2)	N5—C5—C6	110.6 (2)
O3—N8—N2	115.5 (2)	N3—C5—C6	104.5 (2)
O5—N9—O6	127.5 (2)	N5—C5—H5	110.7
O5—N9—N3	116.1 (2)	N3—C5—H5	110.7
O6—N9—N3	116.2 (2)	C6—C5—H5	110.7
O8—N10—O7	126.7 (2)	N6—C6—N4	110.0 (2)
O8—N10—N4	116.4 (2)	N6—C6—C5	108.0 (2)
O7—N10—N4	116.9 (2)	N4—C6—C5	103.7 (2)
O10—N11—O11	125.4 (2)	N6—C6—H6	111.6
O10—N11—N6	117.0 (2)	N4—C6—H6	111.6
O11—N11—N6	117.6 (2)	C5—C6—H6	111.6
N1—C1—N2	95.6 (2)	O9—C7—N5	122.8 (3)
N1—C1—C4	113.2 (2)	O9—C7—H7	118.6
N2—C1—C4	112.7 (2)	N5—C7—H7	118.6
			
C1—N1—N7—O1	−17.6 (3)	C1—N2—C3—N6	88.6 (3)
C2—N1—N7—O1	−157.8 (2)	N8—N2—C3—C2	−165.5 (2)
C1—N1—N7—O2	164.7 (2)	C1—N2—C3—C2	−26.8 (3)
C2—N1—N7—O2	24.5 (3)	N5—C2—C3—N6	−0.7 (3)
C1—N2—N8—O4	−161.3 (2)	N1—C2—C3—N6	−120.0 (2)
C3—N2—N8—O4	−25.1 (3)	N5—C2—C3—N2	118.4 (2)
C1—N2—N8—O3	19.8 (3)	N1—C2—C3—N2	−0.9 (2)
C3—N2—N8—O3	156.0 (2)	N10—N4—C4—N3	112.9 (2)
C4—N3—N9—O5	−35.7 (3)	C6—N4—C4—N3	−32.2 (3)
C5—N3—N9—O5	−164.2 (2)	N10—N4—C4—C1	−132.1 (2)
C4—N3—N9—O6	148.8 (2)	C6—N4—C4—C1	82.8 (2)
C5—N3—N9—O6	20.3 (3)	N9—N3—C4—N4	−100.2 (2)
C4—N4—N10—O8	−163.1 (2)	C5—N3—C4—N4	32.8 (2)
C6—N4—N10—O8	−21.6 (3)	N9—N3—C4—C1	145.4 (2)
C4—N4—N10—O7	20.8 (3)	C5—N3—C4—C1	−81.5 (2)
C6—N4—N10—O7	162.4 (2)	N1—C1—C4—N4	−108.5 (2)
C6—N6—N11—O10	176.8 (2)	N2—C1—C4—N4	−1.5 (3)
C3—N6—N11—O10	13.9 (3)	N1—C1—C4—N3	2.7 (3)
C6—N6—N11—O11	−6.2 (3)	N2—C1—C4—N3	109.7 (2)
C3—N6—N11—O11	−169.1 (2)	C7—N5—C5—N3	117.4 (2)
N7—N1—C1—N2	173.7 (2)	C2—N5—C5—N3	−61.1 (3)
C2—N1—C1—N2	−43.1 (2)	C7—N5—C5—C6	−128.0 (2)
N7—N1—C1—C4	−68.7 (3)	C2—N5—C5—C6	53.5 (3)
C2—N1—C1—C4	74.5 (3)	N9—N3—C5—N5	−131.2 (2)
N8—N2—C1—N1	−178.4 (2)	C4—N3—C5—N5	97.1 (2)
C3—N2—C1—N1	42.3 (2)	N9—N3—C5—C6	110.3 (2)
N8—N2—C1—C4	63.6 (3)	C4—N3—C5—C6	−21.5 (3)
C3—N2—C1—C4	−75.7 (3)	N11—N6—C6—N4	−107.2 (3)
C7—N5—C2—N1	−119.4 (2)	C3—N6—C6—N4	56.2 (3)
C5—N5—C2—N1	59.1 (3)	N11—N6—C6—C5	140.3 (2)
C7—N5—C2—C3	128.1 (2)	C3—N6—C6—C5	−56.4 (3)
C5—N5—C2—C3	−53.4 (3)	N10—N4—C6—N6	118.3 (2)
N7—N1—C2—N5	53.7 (3)	C4—N4—C6—N6	−96.4 (2)
C1—N1—C2—N5	−89.3 (2)	N10—N4—C6—C5	−126.4 (2)
N7—N1—C2—C3	171.6 (2)	C4—N4—C6—C5	18.8 (3)
C1—N1—C2—C3	28.6 (3)	N5—C5—C6—N6	0.6 (3)
N11—N6—C3—N2	108.3 (3)	N3—C5—C6—N6	118.4 (2)
C6—N6—C3—N2	−54.9 (3)	N5—C5—C6—N4	−116.1 (2)
N11—N6—C3—C2	−140.3 (2)	N3—C5—C6—N4	1.7 (3)
C6—N6—C3—C2	56.4 (3)	C5—N5—C7—O9	3.0 (4)
N8—N2—C3—N6	−50.1 (3)	C2—N5—C7—O9	−178.6 (3)
